# Resolvin D1 drives establishment of *Leishmania amazonensis* infection

**DOI:** 10.1038/srep46363

**Published:** 2017-04-10

**Authors:** Hayna Malta-Santos, Bruno B. Andrade, Dalila L. Zanette, Jackson M. Costa, Patrícia T. Bozza, Christianne Bandeira-Melo, Aldina Barral, Jaqueline França-Costa, Valéria M. Borges

**Affiliations:** 1 Instituto Gonçalo Moniz, Fundação Oswaldo Cruz (FIOCRUZ), Salvador, Brazil; 2 Universidade Federal da Bahia, Salvador, Brazil; 3 Multinational Organization Network Sponsoring Translational and Epidemiological Research (MONSTER) Initiative, Fundação José Silveira, Salvador, Brazil; 4 Laboratório de Imunofarmacologia, Instituto Oswaldo Cruz, Fundação Oswaldo Cruz, Rio de Janeiro, Brazil; 5 Instituto de Biofísica Carlos Chagas Filho (IBCCF), Universidade Federal do Rio de Janeiro, Rio de Janeiro, Brazil

## Abstract

Previous studies have indicated that the balance between different eicosanoids reflect the intensity of the inflammatory profile in patients with tegumentary leishmaniasis. More recently, pro-resolution lipid mediators have been shown to play critical roles in dampening pathological inflammatory processes to reestablish homeostasis in a diverse range of experimental settings. Among these lipid mediator, resolvins from D series have been described as potent anti-inflammatory and immunomodulatory mediators, and its activities include inhibition of leukocyte chemotaxis and blockage production of proinflammatory cytokines, while increasing the expression of regulatory mediators. Whether resolvins play significant roles in establishment and persistence of *Leishmania* infection is currently unknown. We addressed this question in the current study by assessing circulating levels of D-series resolvins in tegumentary leishmaniasis patients presenting with localized or diffuse disease. We found heightened expression of resolvin D1 in diffuse cutaneous leishmaniasis which was correlated with expression profile of biomarkers associated with disease pathogenesis. Additional *in vitro* experiments using primary human macrophages indicated that resolvin D1 may promote intracellular *Leishmania amazonensis* replication through a mechanism associated with induction of heme oxygenase-1. These results suggest that targeting resolvin D1 could serve as potential strategy for host directed therapy in diffuse cutaneous leishmaniasis.

Resolvins are oxygenated lipid mediators derived from ω-3 polyunsaturated fatty acids that have been associated with resolution of acute inflammation and restoration of tissue homeostasis^1^. The majority of the studies involving resolvins has focused on those from the D series, represented mainly by resolvin D1 (RvD1) and D2 (RvD2)^1^. The biological effects attributed to resolvins have been linked to its anti-inflammatory and immunomodulatory properties, which include inhibition of leukocyte chemotaxis, blocking production of pro-inflammatory cytokines, while increasing the expression of anti-inflammatory mediators such as heme oxygenase 1 (HO-1)^2^,^3^. The anti-inflammatory activity of resolvins has been extensively described in several inflammatory disease models, including cardiovascular diseases, cancer, acute kidney and lung injuries and metabolic inflammation in adipose tissue^1^,^3^. More recently, resolvins have also been implicated in host protective responses during viral and bacterial infections^1^. Although lipid mediator levels in plasma have been reported in patients with tegumentary leishmaniasis^4^, it is still unknown whether resolvins associate with disease pathogenesis driven by *Leishmania* infection.

Tegumentary leishmaniasis is a disease caused by *Leishmania* parasites and exhibits a spectrum of clinical manifestations associated with the balance between parasite replication and immune-mediated inflammatory destruction of the skin tissue^5^. In the most common clinical form, named localized cutaneous leishmaniasis (LCL), single self-healing skin ulcers are usually observed. In this setting, a modest infiltration of macrophages is detected, with very few parasites, due moderate inflammation and cell-mediated immune responses^5^. A rare clinical form, diffuse cutaneous leishmaniasis (DCL), is characterized by the numerous nonulcerated nodular lesions with heavily parasitized macrophages^6^. Patients with DCL lack protective cell-mediated immunity and are reported to present elevated expression of anti-inflammatory mediators such as TGF-β and arginase-I while exhibiting reduced circulating levels of TNF-α and IL-12p70^7^, highlighting a relative state of immunosuppression. Probably for this reason, DCL disease continues to develop for decades and exhibits treatment resistance and relapse^6^.

*In vitro* experiments have demonstrated that during *Leishmania* infection, there is increased production of arginase-I, transforming growth factor β (TGF-β) and HO-1 by infected macrophages, which has been associated with augmented intracellular parasite proliferation^7^,^8^,^9^,^10^. Moreover, the role of these three important mediators in *Leishmania* infection has been further established in experiments using macrophages from mice genetically lacking HO-1^11^, and pharmacological inhibition of arginase-I^7^ or antibody-mediated blockage of TGF-β^12^ in infected human macrophages. In all these experimental settings, reduction of HO-1, arginase-I and TGF-β resulted in substantial increase in anti-parasite effector mechanisms and production of inflammatory cytokines such as TNF-α, resulting in better control of parasite loads *in vitro*^7^,^11^,^12^. Whether resolvins play any role in these phenomena in the context of *L. amazonensis in vitro* or *in vivo* is unknown.

Here, to characterize the role of RvD1 and RvD2 in *Leishmania* infection, we assessed circulating levels of these lipid mediators in patients with LCL or DCL from an endemic area in Brazil. We observed that RvD1 concentrations in plasma samples from DCL patients were substantially increased compared to those with LCL and that its levels were positively correlated with arginase-I and TGF-β, while being negatively correlated with TNF-α levels. In addition, we performed *in vitro* assays with primary monocyte-derived human macrophages infected with *L. amazonensis.* Notably, *L. amazonensis* infection of macrophages primed RvD1 production and its supplementation to cultures amplified intracellular parasite replication, arguing that RvD1 may promote parasite persistence. The findings presented here, which still need further validation in different patient populations and epidemiological settings, point to the idea that interfering with the RvD1 pathway could potentially serve as an adjunctive therapy for DCL.

## Results

### RvD1 plasma levels are associated with an immune profile that distinguishes patients with DCL from those with LCL

**Figure 1:**
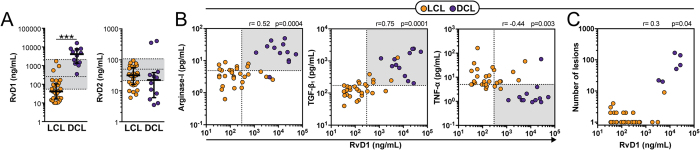
Differential plasma concentrations of RvD1 and RvD2 in patients with tegumentary leishmaniasis. (**A**) Serum levels of RvD1 and RvD2 in patients with localized cutaneous leishmaniasis (LCL; n = 29) or diffuse cutaneous leishmaniasis (DCL; n = 12). Shaded area represent median and interquartile range values obtained in 42 healthy endemic controls to serve as reference. Data were compared using the Mann-Whitney *U* test (***P < 0.0001). (**B**) Correlations between plasma RvD1 levels and arginase-I, transforming growth factor-β1 (TGF-β) and tumor necrosis factor-α (TNF-α) in active LCL and DCL patients. Dotted lines on the X-axis represent the median value of RvD1 within the entire study population whereas dotted lines on each Y-axis indicate median values for each mediator. Gray areas designate the quadrants that include the individuals simultaneously displaying values of RvD1 and madiators above the medians or in the case of TNF-α below the median. (**C**) Correlation between plasma RvD1 levels and number of lesions in the study population. Correlations were tested using Spearman ranks.

Patients with DCL displayed at least 100-fold higher levels of RvD1 in plasma compared with those with LCL (Fig. 1A). Concentrations of RvD2 were not different between these clinical groups (Fig. 1A). These findings made us hypothesize that RvD1 levels could be associated with an immunological environment that favors the development of DCL. A recent study reported that DCL patients present heightened levels of arginase-I and TGF-β whereas those with LCL display increased plasma concentrations of TNF-α^7^. We next tested if RvD1 levels are associated with this immune profile. RvD1 levels in plasma exhibited strong positive correlations with concentrations of arginase-I and TGF-β and were negatively correlated with TNF-α levels (Fig. 1B). To test if the high plasma levels of RvD1 is associated with increased *Leishmania* replication *in vivo*, we performed additional analyses, which revealed that RvD1 levels were positively correlated with the number of lesions in the study population (Fig. 1C). These observations suggested that RvD1 but not RvD2 may be differentially implicated in the pathogenesis of LCL and DCL and that RvD1 could be associated with the dampened inflammation observed in DCL, which favors *Leishmania* replication.

### RvD1 enhances *L. amazonensis* infection burden in human macrophages

**Figure 2:**
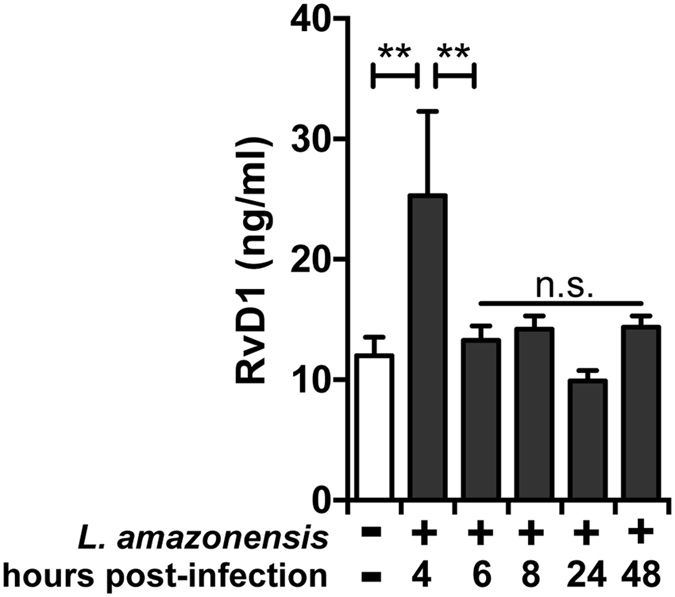
*L. amazonensis* infection induces the RvD1 production by macrophages. Monocyte-derived human macrophages (n = 6) were infected with *L. amazonensis* (MOI 6:1). RvD1 levels were measured in culture supernatants at indicated timepoints post-infection. Data shown are mean and SE of one representative out two independent experiments. *P < 0.05, **P < 0.01.

To better explore the direct association between RvD1 and *Leishmania* infection, we employed an *in vitro* system using primary human monocyte-derived macrophages. In cultures of macrophages infected with *L. amazonensis*, we observed a >1.5-fold induction of RvD1 concentrations in supernatants at 4 h post-infection compared to that in uninfected cell cultures (Fig. 2). This infection-driven induction in RvD1 production was transient as the detected concentrations significantly dropped at as soon as 6 h post infection and persisted for up to 48 h at low levels similar to pre-infection status (Fig. 2).

**Figure 3:**
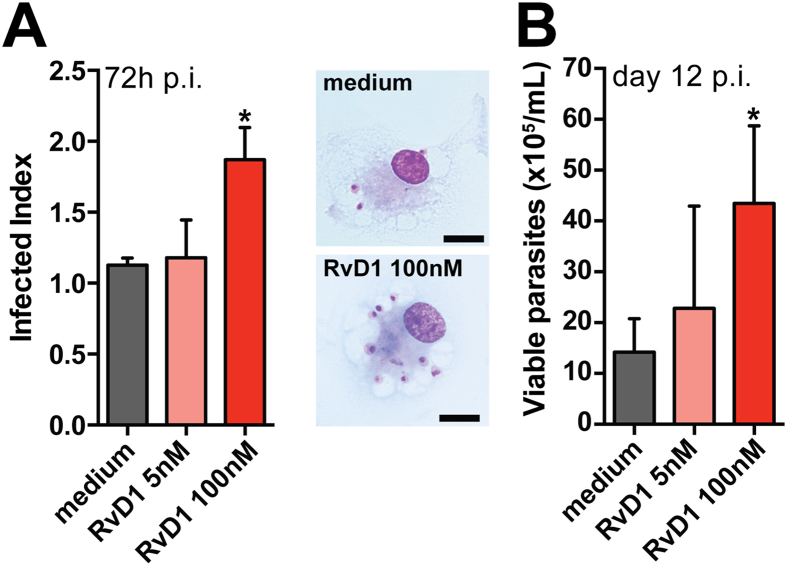
Effectiveness of Resolvin D1 supplementation in cultures of *L. amazonensis*- infected human macrophages. Monolayers of infected macrophages were cultured with medium alone or with indicated doses of synthetic RvD1. (**A**) Intracellular infection burden was assessed by light microscopy; micrographs from *L. amazonensis*-infected human macrophages unstimulated or treated with RvD1 for 72 h. Original magnification x 1000. (**B**) Counting of viable parasites was performed as described in Methods. Data represent mean and SE of one representative out two independent experiments. *P < 0.05, **P < 0.01.

To mimic an environment with high RvD1 levels such as that observed in DCL patients, we supplemented cultures of macrophages infected with *L. amazonensis* with increasing doses of this lipid mediator. We found an increased infection index as well as augmented number of viable replicating parasites inside macrophages treated with 100 nM RvD1 (Fig. 3A). Photomicrographs illustrated that treatment with RvD1 was able to increase intracellular parasite burden more efficiently than cells in the untreated group (Fig. 3A).

**Figure 4:**
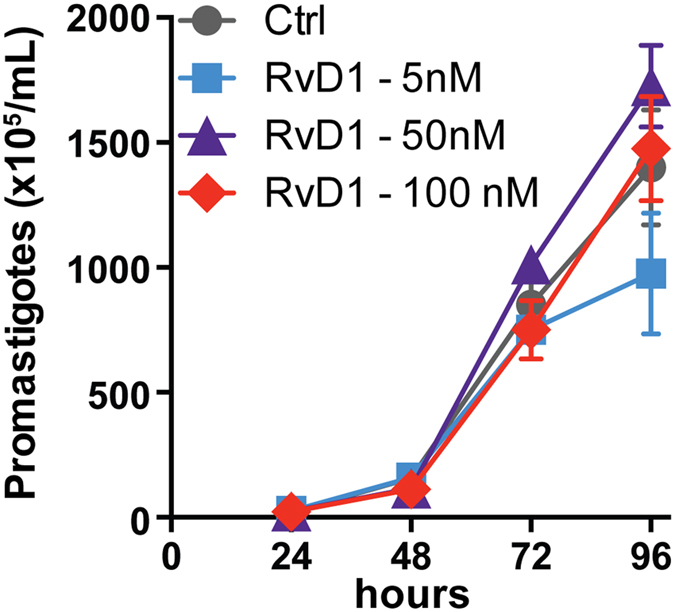
Effect of Resolvin D1 (RvD1) in axenic *L. amazonensis* cultures. Parasites were incubated for 4 days with medium alone (Ctrl) or with indicated doses of RvD1. The number of viable parasites was evaluated by direct counting. Each point represents mean and SE. Data are representative of at least 3 independent assays and were collected in triplicate for each condition.

To assess if RvD1 could be exerting an effect in the parasite growth or survival *in vitro* we treated axenic *L. amazonensis* promastigote cultures with increasing doses of RvD1. We found that RvD1 supplementation in parasite cultures did not affect the growth curves (Fig. 4) These results are consistent with the idea that the parasite triggers an early and transient production of RvD1 by macrophages which should be important for the *Leishmania* intracellular proliferation.

### RvD1 supplementation amplifies *L. amazonensis* driven HO-1 production in infected macrophages

**Figure 5:**
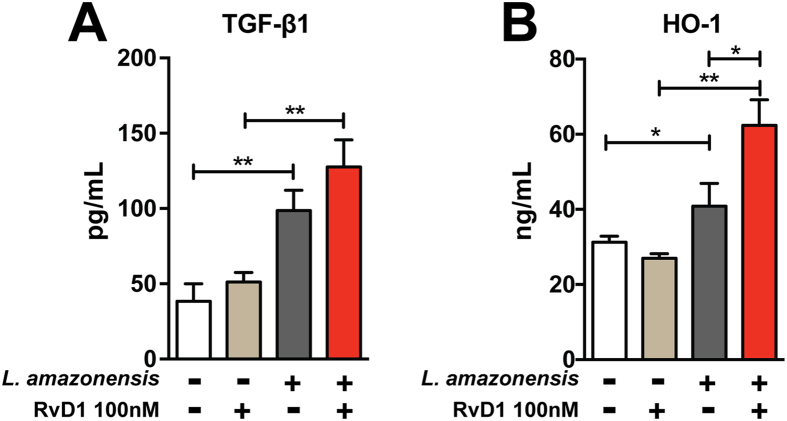
RvD1 induces the HO-1 production in *L. amazonensis*-infected human macrophages. Supernatants from infected macrophages were collected after 24 h post infection and evaluated for the levels of transforming growth factor β (TGF-β; **A**) whereas hemoxygenase-1 (HO-1; **B**) protein expression was assessed in cell extracts as described in Methods. Data shown are mean and SE of one representative out two independent experiments. *P < 0.05, **P < 0.01.

It is well established that induction of cytokines is a critical evasion mechanism used by *Leishmania* parasites^10^,^13^. Here, we confirmed that *L. amazonensis* infection induces TGF-β production by infected macrophages, as previously reported^14^ (Fig. 5A). Interestingly, treatment of cultures with RvD1 was not able to further increase TGF-β levels in this *in vitro* system (Fig. 5A). Furthermore, we found no differential induction of arginase activity and TNF-α expression in cultures treated with RvD1 (data not shown).

An important mechanism used by *Leishmania* parasites to subvert host antimicrobial responses is the induction of antioxidants^11^,^15^. In the present study, *L. amazonensis* infection of macrophages induced HO-1 expression similarly to a previous observation in *L. chagasi* infected cells^11^. Notably, RvD1 supplementation induced substantially higher expression of HO-1 in cell lysates compared to untreated cultures (Fig. 5B). Despite these differences observed *in vitro*, we found that plasma levels of HO-1 were undistinguishable between LCL and DCL patients (data not shown).

### Pharmacological treatment of macrophage cultures with Baicalein reduces intracellular *L. amazonensis* infection

**Figure 6:**
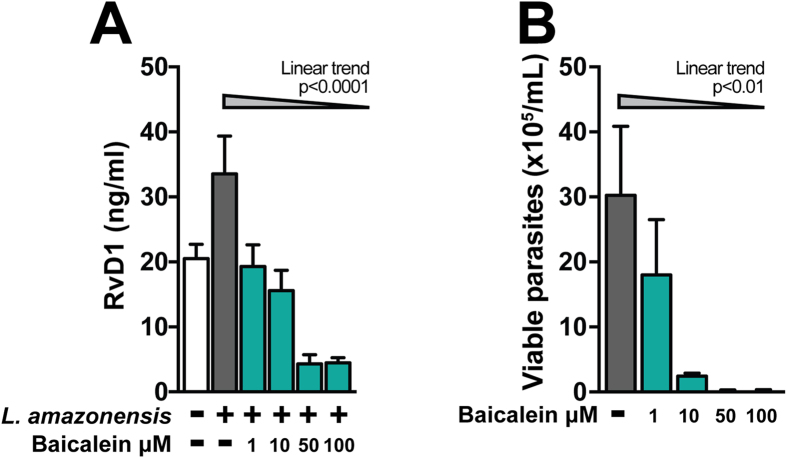
Baicalein inhibits the *L. amazonensis* proliferation. The effect of 15-lipoxygenase in *L. amazonensis* intracellular growth was examined by treating infected macrophage cultures with indicated doses of Baicalein. (**A**) RvD1 levels were measured in culture supernatants 4 h post infection. (**B**) Counting viable parasites was assessed in cells treated with increasing doses of baicalein. Data shown are mean and SE of one representative out two independent experiments. *P < 0.05, **P < 0.01.

To further delineate the direct link between intrinsic RvD1 induction and *L. amazonensis* replication, we treated infected macrophages with Baicalein, an inhibitor of 15-lipoxygenase^16^, which is a key enzyme involved in the production of RvD1^17^. Treatment with Baicalein resulted in a dose dependent reduction of RvD1 production at 4 h post-infection (Fig. 6A). This reduction was associated with a significant decrease in intracellular parasite viability (Fig. 6B). These findings strongly argue that blocking RvD1 production may serve as a strategy to reduce intracellular *L. amazonensis* infection burden.

## Discussion

Resolvins are derived from ω-3 fatty acid and represent a new class of lipid mediator^1^. Studies have demonstrated that resolvins play a pivotal role during the resolution phase of acute inflammation and restoration of tissue homeostasis^18^,^19^. Its biological effects have been described in several inflammatory diseases^17^ as well as in viral or bacterial infections^1^. However, the role of resolvins in leishmaniasis is largely unexplored.

In the present study, we show that plasma levels of RvD1, but not of RvD2, were substantially higher in DCL patients compared with that detected in LCL patients. These results suggest that distinct resolvins from the D series may be differentially implicated in the pathogenesis of LCL and DCL. Corroborating with the idea that RvD1 has been shown to modulate inflammatory responses^1^, our correlation analyses revealed that circulating levels of RvD1 exhibited strong positive associations with concentrations of arginase-I and TGF-β, while negatively correlating with TNF-α levels. Previous data from our group in plasma samples from the same patient population have shown high levels of arginase-I and TGF-β, while reduced TNF-α concentrations in DCL patients compared to uninfected controls or individuals with LCL^7^. This study has further demonstrated that *in situ* arginase-I expression was increased in lesions from DCL vs. those from LCL patients. The association between high levels of RvD1 and arginase-I and TGF-β could be ultimately contributing to suppression of host immune responses observed in DCL patients^7^,^20^ and can potentially be critical to disease progression.

In active disease, DCL patients present with disseminated nodular lesions and high intracellular parasite burden in tissue macrophages^21^. Our *in vitro* experiments revealed that RvD1 is associated with increased parasite replication in macrophages. In addition, we found that DCL patients presenting with the highest numbers of cutaneous lesions were the same individuals exhibiting the highest plasma levels of RvD1. These results argue that high RvD1 expression is associated with the anti-inflammatory profile observed in DCL patients, which in turn reflects parasite replication and disease progression. Additional prospective studies are warranted to answer whether RvD1 levels directly affects disease activity in DCL.

It has been demonstrated that resolvins are produced rapidly during the inflammatory response, but have a prolonged effect in different experimental models^18^,^22^. Our study expands the current knowledge in the context of parasite infection, as we demonstrate that RvD1 is transiently induced in macrophages during the first 4 hours upon exposure to *L. amazonensis* but its biological effect in promoting intracellular parasite replication persists up to 72 h post-infection. Our observations suggest that initial RvD1 production by infected macrophages may be crucial to promote *L. amazonensis* intracellular survival and proliferation. Although we demonstrate that *Leishmania* is capable of inducing the RvD1 production in our experimental *in vitro* system, the specific parasite component triggering activation of signaling pathways that drive RvD1 production are yet to be defined.

To our knowledge, no previous study has described the role of RvD1 in *L. amazonensis* proliferation. Our data clearly demonstrated treatment with heightened doses of this lipid mediator was able to increase intracellular parasite loads. Interestingly, treatment of axenic cultures of synthetic RvD1 did not alter the growth curves of the parasite, indicating that the biological effect of this lipid mediator in our experimental system relies on macrophage function rather than on the parasite itself. We then hypothesized that the mechanisms linking macrophage exposure to RvD1 and enhanced intracellular parasite replication could involve subversion of anti-parasite macrophage effector functions. During the *Leishmania* infection, it is well established that parasite can present a series of escape mechanisms, such the deactivation of macrophages^23^, induction of antioxidants^11^,^15^ and anti-inflammatory cytokines, such TGB-β^14^. In other experimental models, resolvins have been shown to induce high levels of TGF-β, increasing phagocytosis of cells by macrophages and inducing an anti-inflammatory phenotype^24^. Herein, we found that *Leishmania* infection triggered the production of TGF-β by macrophages, but RvD1 supplementation in cultures failed to amplify such effect. This result argues that RvD1 may affect another mechanism aside from TGF-β production by macrophages, which may result in increased parasite replication. Notably, TGF-β can be produced by many other cell types^25^,^26^ and thus it is possible that the positive correlation between RvD1 and TGF-β plasma levels observed in our patient population could result from an effect in cells other than macrophages. Future studies investigating the role of RvD1 on other cell types in the context of *Leishmania* infection are needed to better understand these discrepancies between the *in vivo* and *in vitro* systems.

In addition to its anti-inflammatory and pro-resolution effects^1^, RvD1 has been reported to potently reduce oxidative stress damage through induction of HO-1^2^,^27^. In the context of *Leishmania* infection, we have demonstrated that parasite-driven HO-1 production represents an important escape mechanism, which works through modulation of reactive oxygen species production by infected human and mouse macrophages^11^. Here we showed that RvD1 supplementation in macrophage cultures resulted in robust induction of HO-1 protein expression at 24 h post *L. amazonensis* infection. We suggest that aside from the induction of HO-1, there may be additional mechanisms driving the effects of RvD1 on parasite replication. HO-1 is an intracellular enzyme and thus its circulating levels may not directly represent *in situ* expression. Indeed, we found no associations between plasma levels of RvD1 and of HO-1 in tegumentary leishmaniasis patients. We are currently performing studies in additional patients to examine *in situ* expression of HO-1 pathway in DCL patients. Our results are associative rather than definitive and thus investigations depicting other potential mechanisms explaining the effects of RvD1 on intracellular *Leishmania* infection are necessary to improve our knowledge about the modulation of macrophage effector functions by this pro-resolvin lipid mediator.

The role of TNF-α and its relationship with oxidative responses during parasite infections has been described in different models^28^,^29^. Previous work from our group has shown that downregulation of TNF-α production is a key event linking HO-1 driven increased survival of *L. infantum chagasi* in infected macrophages^11^. More recently, a study using peripheral blood mononuclear cells (PBMCs) from patients with Chagas Disease-associated cardiomyopathy demonstrated that RvD1 supplementation in cell cultures interfered with cellular survival without affecting TNF-α production^30^, suggesting that the biological action of RvD1 is not directly linked to TNF-α production. The results presented here, showing no link between RvD1-associated HO-1 induction and changes in TNF-α production, reinforce the idea that RvD1 may act independent of TNF- α in our infection model. The effects of RvD1 and HO-1 on TNF-α production may differ according to the infection model or parasite species, and additional studies with other *Leishmania* species are necessary to confirm this hypothesis.

The inhibition of enzymes from the lipid mediators pathways has been widely used to efficiently control inflammatory responses^31^ as well as *Leishmania* infection burden^32^. In our experimental model, we found that treatment with an inhibitor of 15-lypoxigenase resulted in a reduction of RvD1 production, which mirrored a decrease in intracellular parasite viability. These data suggest that pharmacological inhibition of RvD1 production may serve as a strategy to reduce *L. amazonensis* infection burden.

Our study has some limitations. Quantifying resolvin levels in plasma of patients may not accurately represent *in situ* responses. In addition, our clinical study was cross-sectional and we believe that prospectively assessing RvD1 concentrations at different time points upon initiation of leishmanicidal treatment may give important insights on whether the levels of RvD1 reflect diseases activity. Larger cohort studies from our group are underway to address this question. We had no access to skin biopsy specimens from the study population, and for this reason we could not examine the expression of enzymes related to RvD1 production *in situ* or perform correlations with parasite loads in the skin. Regardless, the positive correlation between RvD1 levels in plasma and number of lesions in the context of DCL (which is indirectly linked to parasite burden *in vivo*) argues that RvD1 production is closed associated with increased parasite replication. Although our *in vitro* experiments clearly demonstrated a relevant biological effect on infected human macrophages, future investigation is necessary to clarify the role of RvD1 in the parasite persistence.

Heightened circulating levels of RvD1 detected in patients with DCL together with the observed strong relationships with biomarkers closely related to an anti-inflammatory environment strongly indicate that this lipid mediator participates in DCL pathogenesis. Furthermore, our *in vitro* results suggest that RvD1 may induce anti-oxidative mechanisms in *L. amazonensis*-infected macrophages, which cause these cells to become more permissive to parasite infection and proliferation.

## Methods

### Ethics Statement

Written informed consent was obtained from all participants or their legally responsible guardians, and all clinical investigations were performed according to the principles expressed in the Declaration of Helsinki. The clinical protocols from which the plasma samples were used in the present study were approved by Institutional Review Board of Instituto Gonçalo Moniz, Fundação Oswaldo Cruz, Brazil (license number 136/2007).

### Patients

The present study used cryopreserved EDTA plasma samples from age- and sex- matched patients with LCL (n = 29, male to female ratio, 1.9; mean age [±standard deviation, SD], 34 ± 15 years) and those with DCL (n = 12, male to female ratio, 1.4; mean age [±SD], 23 ± 17 years). Patients with LCL were recruited at our reference clinic in Jiquiriçá, Bahia, Brazil. Patients with DCL were from Maranhão State. LCL patients were recruited at the time of disease presentation, were treatment naïve and had no previous diagnosis of tegumentary leishmaniasis. Clinical and epidemiological characterization, as well as the diagnostic approaches, have been reported previously^7^. To serve as a reference for the plasma assays, we have included plasma samples from 42 age and gender matched healthy endemic controls, who were family members of the patients with DCL, exhibiting no cutaneous lesions or prior CL history and negative DTH response.

### Immunoassays

Concentrations of RvD1 and RvD2 were measured using an enzyme-linked immunoassay (Cayman Chemical, Ann Harbor, MI) according to the manufacturer’s instruction. TGF-β, TNF-α (R&D Systems, Minneapolis, Minnesota) and RvD1 (Cayman Chemical) expression was measured in supernatants cells according to the manufacturer’s protocols. The expression of heme oxygenase-1 (HO-1) in cell lysates were measured an ELISA kit (Enzo Life Sciences, NY) by the protocols.

### Cell Culture

Column purified CD14^+^ monocytes were obtained from buffy coats from healthy blood donors (from the Hematology and Hemotherapy Foundation of Bahia, HEMOBA), and plated at 2 × 10^6^ cell/well in 24-well plates containing RPMI with 10% fetal bovine serum and treated for 7 d with 50 ng/mL MCSF (Peprotech, Rock Hill, NJ) to differentiate in human macrophages as previously described^33^. Macrophages were infected (multiplicity of infection 6:1) with stationary phase *L. amazonensis* promastigotes (MHOM/BR/87/BA336) isolated from a patient with DCL. After 4 h incubation at 37 °C, free parasites were removed by extensive washing with phosphate-buffered saline. Then, indicated concentrations of synthetic RvD1 (Cayman Chemical), with or without Baicalein (a 15-LO inhibitor, TOCRIS, Bristol, UK) were added to cultures. Culture supernatants were collected after 24 h post infection for measurement inflammatory mediators as described above. The intracellular parasite loads were quantified by microscopy and viable promastigotes in Schneider medium. The infectivity index (percentage of infected macrophages x average number of amastigote per macrophage) was determined by randomly counting at least 200 macrophages per slide in light microscope, using the immersion objective (100x).

### *In vitro* treatments

Axenic cultures of *L. amazonensis* promastigotes were incubated at 26 °C in Schneider’s complete medium (Invitrogen) supplemented with 10% inactive Fetal Bovine Serum (FBS), 2 mM L-glutamine, 100 U/ml penicillin, and 100 mg/ml streptomycin (all from Invitrogen). For assessment of a potential direct biological effect of resolvins on parasite cultures, promastigotes in stationary phase (2 × 10^5^/mL) were cultured with supplemented Schneider medium alone or in combination with indicated concentrations of RvD1 or RvD2 (5, 50 and 100 nM). Cultures were incubated for 4 days at 24 °C and the effect of these drugs on parasite growth was evaluated by directing counting daily live motile parasites using a Neubauer chamber.

### Statistical analysis

Median and interquartile ranges were used as measures of central tendency for the *ex vivo* analyses. Mean and standard errors were used to display data from the *in vitro* experiments. Differences between groups were calculated using the Mann–Whitney *U* test (2-groups) or the Kruskal-Wallis test with the Dunn multiple comparisons or linear trend analysis post tests (more than 2 groups). Correlations were tested using Spearman ranks. Differences with p-values < 0.05 were considered statistically significant.

## Additional Information

**How to cite this article**: Malta-Santos, H. *et al*. Resolvin D1 drives establishment of *Leishmania amazonensis* infection. *Sci. Rep.* **7**, 46363; doi: 10.1038/srep46363 (2017).

**Publisher's note:** Springer Nature remains neutral with regard to jurisdictional claims in published maps and institutional affiliations.
